# Spin-Forbidden Addition of Molecular Oxygen to Stable Enol Intermediates—Decarboxylation of 2-Methyl-1-tetralone-2-carboxylic Acid

**DOI:** 10.3390/ijms24087424

**Published:** 2023-04-18

**Authors:** Pablo Ortega, Sara Gil-Guerrero, Lola González-Sánchez, Cristina Sanz-Sanz, Pablo G. Jambrina

**Affiliations:** 1 Departamento de Química-Física, Universidad de Salamanca, 37008 Salamanca, Spain; 2 CICECO—Aveiro Institute of Materials, University of Aveiro, 3810-193 Aveiro, Portugal; 3 Departamento de Química Física Aplicada, Universidad Autónoma de Madrid, 28049 Madrid, Spain

**Keywords:** intersystem crossing, peroxidation, minimum-energy-crossing-point

## Abstract

The deprotonation of an organic substrate is a common preactivation step for the enzymatic cofactorless addition of O${}_{2}$ to this substrate, as it promotes charge-transfer between the two partners, inducing intersystem crossing between the triplet and singlet states involved in the process. Nevertheless, the spin-forbidden addition of O${}_{2}$ to uncharged ligands has also been observed in the laboratory, and the detailed mechanism of how the system circumvents the spin-forbiddenness of the reaction is still unknown. One of these examples is the cofactorless peroxidation of 2-methyl-3,4-dihydro-1-naphthol, which will be studied computationally using single and multi-reference electronic structure calculations. Our results show that the preferred mechanism is that in which O${}_{2}$ picks a proton from the substrate in the triplet state, and subsequently hops to the singlet state in which the product is stable. For this reaction, the formation of the radical pair is associated with a higher barrier than that associated with the intersystem crossing, even though the absence of the negative charge leads to relatively small values of the spin-orbit coupling.

## 1. Introduction

Photodynamic therapy (PDT) is a clinically approved technique that has shown great potential for the treatment of certain kinds of tumours [1,2,3]. In PDT, a photosensitizer is introduced into the organism, where it is photo-excited to a singlet excited electronic state, followed by its relaxation via intersystem crossing to a triplet state. Once in the triplet state, the photosensitizer interacts with molecular oxygen, leading to the formation of singlet oxygen (or another reactive oxygen species), a cytotoxic molecule [4,5,6] that can induce the apoptosis and necrosis of tumour cells, damage the tumour-associated vasculature, or induce an immune response [7].

The production of singlet O${}_{2}$ from its triplet ground state is not efficient, as this transition is forbidden by the spin, parity, and angular momentum selection rules for the electric dipole process [8], as is evinced by the long lifetime of singlet O${}_{2}$ in the gas phase (∼1 h) [9]. The drastic difference in reactivity between the ground state (triplet) O${}_{2}$ and the singlet O${}_{2}$ is caused by the nature of the electronic ground state. While most organic molecules are closed-shell (singlet) molecules, O${}_{2}$ is a diradical, and so reactions between them are spin-forbidden.

To catalyze spin-forbidden reactions with O${}_{2}$, enzymes typically rely on metal cofactors, which can easily switch the total spin of the system [10,11,12,13,14,15,16,17]. Others, particularly flavoenzymes, use a non-metal cofactor, or require no cofactor at all [18,19,20,21,22,23,24,25], and although there are some common features, such as the activation of the organic substrate via deprotonation [20], it is not clear regarding the role played by enzymes to catalyze spin-forbidden reactions. Electronic structure calculations have been used to unravel the mechanisms of these reactions (see, for example, Refs. [26,27,28,29,30,31,32,33,34,35]), but there is no consensus mechanism. For example, the reaction catalyzed by glucose oxidase needs a proton donor (His516) [31], whereas for the DpgC-catalyzed reaction, peroxidation occurs without charged residues or water molecules that could act as a base [27,36].

To investigate spin-forbidden processes using computational methods, non-adiabatic Transition State Theory (NA-TST) is one of the most powerful frameworks [37,38,39]. In NA-TST, the minimum energy crossing point (MECP) between the two electronic states with different multiplicities plays the same role as the transition state in “adiabatic TST”, so that its energy has to be determined. However, overcoming the MECP is not sufficient for the reaction to proceed. In NA-TST, the transmission coefficient depends on the hopping-probability, which ultimately depends on the value of the spin-orbit coupling (SOC) [40], the non-relativistic quantum effect that permits transitions between PESs of different multiplicities. Hence, the spin-forbidden character of the reaction acts as an additional barrier [38], and only those processes for which the value of SOC is not negligible could proceed at a reasonable speed. SOC strength depends strongly on the nature of the two states involved in the spin-change process. According to El-Sayed’s rules [41], SOC is expected to be larger when the change of spin is associated with a change in the orbital angular momentum (or electronic configuration). For collisions involving O${}_{2}$, it implies that the ability of the solvent or organic molecules to promote O${}_{2}$ electronic transitions is associated with the perturbation of the symmetry of O${}_{2}$, even if they only interact via weak Van der Waals forces [8,42,43]. Recent studies have suggested a correlation between the value of SOC and the energy difference between the two π orbitals for complexes involving O${}_{2}$ [43]. On top of the importance of the SOC value, according to the Landau-Zenner equation [39,44], the hopping probability also depends on the reduced mass associated with the vibrational coordinate parallel to the crossing seam, and the difference of the gradient between the two PESs involved [45].

**Figure 1:**
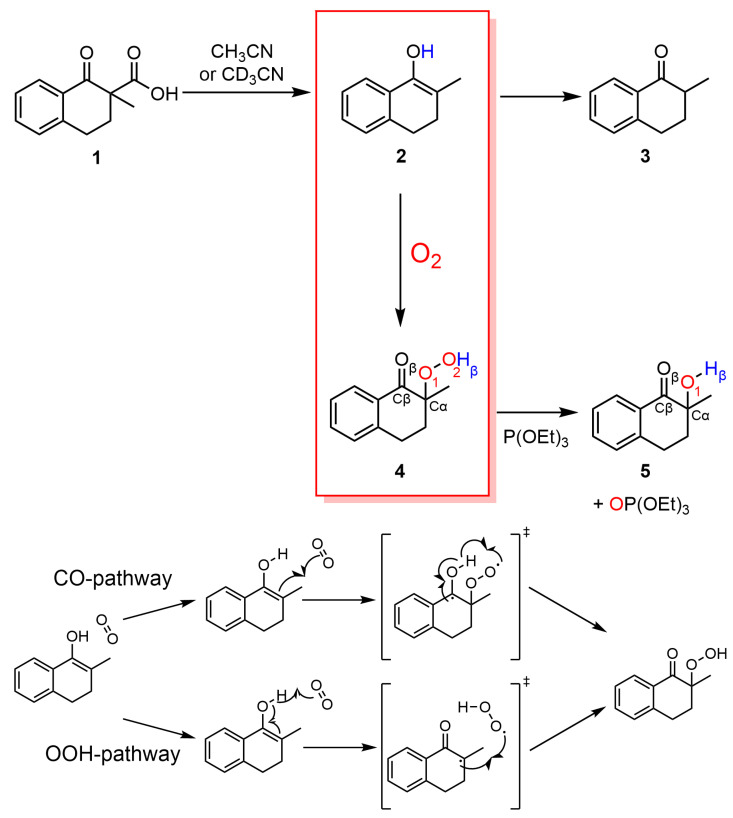
Top panel: Decarboxylation of 2-methyl-1-tetralone-2-carboxylic acid (**1**) involves the formation of an enol intermediate (**2**) [51]. This enol is affected by a side reaction with atmospheric oxygen to obtain the hydroperoxide tetralone-derivative (**4**). The reduction of (**4**) using P(OEt)${}_{3}$ leads to the α-ketol (**5**). The transferred proton is colored in blue, and atoms are labeled according to the notation used in the article. Bottom panel: Possible reaction pathways for the peroxidation of the 2-methyl-3,4-dihydro-1-naphthol molecule. CO-pathway (upper path), where the Cα-O1 bond is formed first, and then the peroxide is protonated, and the OOH-pathway, in which the protonation of O${}_{2}$ occurs prior to the formation of the CαO1 bond.

Ground state (triplet) O${}_{2}$ is also used as an oxidant for many synthetic pathways (see, for example, Refs. [46,47,48,49,50,51,52,53,54,55,56,57] and references therein) as it is a very abundant and environmentally friendly reagent. To circumvent the limitation imposed by the spin-forbidden characters of these reactions, most of them use metal centers as catalyzers. One example of the use of triplet O${}_{2}$ in the absence of metal cofactors is that proposed by Riahi et al. [51], who reported on the formation of a hydroperoxide (2-hydroperoxy-2-methyl-1-tetralone (**4**)) in the decarboxylation of 2-methyl-1-tetralone-2-carboxylic acid (**1**) in acetonitrile, as depicted in Figure 1. Under aerobic (and dark) conditions, O${}_{2}$ reacts with a stable enol intermediate (**2**), leading to the formation of the hydroperoxide that was isolated and subsequently reduced with P(OEt)${}_{3}$, leading to the formation of an α-ketol (**5**). Based on unrestricted DFT calculations, Riahi et al. [51] postulated a mechanism in which the system first overcomes a barrier in the triplet state, after which it undergoes a transition from the triplet to the singlet state, which will evolve to the formation of the hydroperoxide. There are some reasons for why the study of the mechanism of this reaction is particularly interesting. First, the addition of O${}_{2}$ to the double bond resembles the kind of processes that occur in the enzymatic additions of O${}_{2}$. Second, unlike most cofactorless enzymatic additions of O${}_{2}$, the process does not require prior deprotonation of the substrate. Then, this study might shed light on why prior deprotonation occurs in biological media. Three, the process involves both the formation of the peroxide bond and hydrogen (or proton) transfer from the substrate to the O${}_{2}$ moiety. Deciphering the sequence of events could help us to advance toward a consensus mechanism for the cofactorless addition of molecular oxygen to organic molecules. Finally, from a computational point of view, this is a somewhat small system, and so higher-level electronic structure methods such as CASPT2 (multi-reference) or Coupled-Cluster (single-reference) methods could be applied.

In this manuscript, we have carried out a comprehensive computational study of the formation of 2-hydroperoxy-2-methyl-1-tetralone using DFT, CASPT2, and Coupled-Cluster calculations. Two different pathways will be evaluated (see the bottom panel of Figure 1), that proposed by Riahi et al. [51], hereinafter, the CO-pathway, for which the peroxide bond is formed prior to ISC, and a second mechanism, the OOH-pathway, in which the first step is the protonation of the O${}_{2}$ moiety. Our results show that the OOH-pathway is preferred, although the barrier for both mechanisms is similar, suggesting that both mechanisms are feasible for similar reactions.

## 2. Results and Discussion

**Figure 2:**
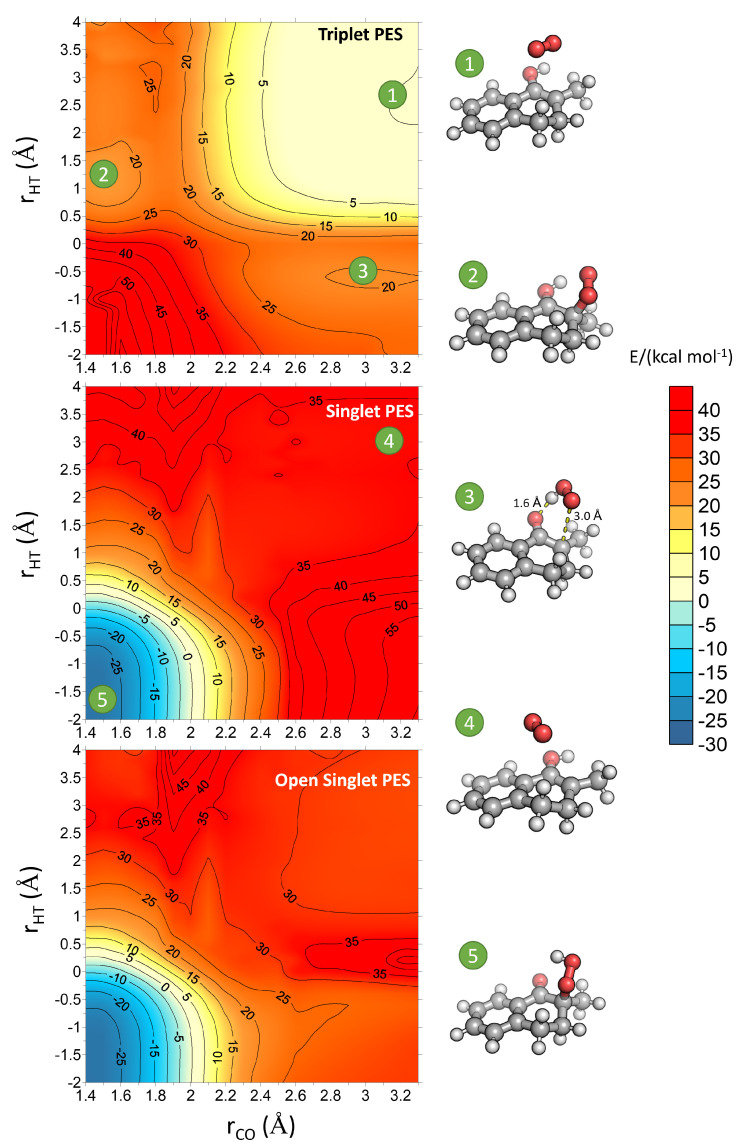
Three-dimensional contour maps of the energy profile for the addition of O${}_{2}$ to 2-methyl-3,4-dihydro-1-naphthol as a function of the Cα-O1 distance and r${}_{\mathrm{HT}}$ = r${}_{O2-H\beta }$− r${}_{O\beta -H\beta }$. Geometries were optimized for their corresponding spin state. Top panel: Lowest triplet state. Middle panel: Lowest closed-shell singlet state. Bottom panel: Lowest open-shell singlet state. The relevant geometries are depicted on the right and labeled over the contour map (see main text). Energies are expressed in kcal/mol, following the color scheme shown in the scale, and distances are expressed in Å. Calculations were carried out at the M062X-D3/maug-cc-pVDZ level of theory. Cartesian coordinates corresponding to structures #1–#5 are shown in Table S1. The zero of the energy scale corresponds to the reactants complex.

Figure 2 displays the energy contour plots, calculated at the M062X-D3/maug-cc-pVDZ level of theory, for the approach of O${}_{2}$ to the enol on the singlet (restricted and open-singlet) and triplet PESs as a function of r${}_{\mathrm{CO}}$, the distance between C${}_{v\alpha }$ and the closest O atom of O${}_{2}$, and r${}_{\mathrm{HT}}$, the reaction coordinate for the proton transfer from the enol to O${}_{2}$ (see Methods section for details). On the triplet state (top panel), we observed the minimum corresponding to the reactants’ asymptote at r${}_{CO}$ = 3.2 Å, and with H${}_{\beta }$ bonded to C${}_{\beta }$ ($r_{\mathrm{HT}}$ > 0) (Structure #1). We also observe two local minima that are considerably higher in energy (17–19 kcal/mol above the reactants asymptote, see Table 1), corresponding to the deprotonated peroxide (Structure #2) and with the protonated ·O${}_{2}$-H radical (Structure #3). To reach any of these secondary minima, the system has to surmount electronic barriers of about 22 kcal/mol (see Table 1). It is worth noticing that there are no minima corresponding to the protonated peroxide, which would appear at the bottom-left corner of the figure (negative values of $r_{\mathrm{HT}}$ and small values of r${}_{\mathrm{CO}}$).

On the contrary, on the singlet PES (middle panel of Figure 2), we observed one deep minimum (Structure #5) that corresponds to the protonated peroxide, 27.8 kcal/mol below the reactants asymptote. No minima associated with either the deprotonated peroxide or the ·O${}_{2}$-H radical appear. The energy difference between the singlet and the triplet PES at the reactants asymptote (Structure #1 and #4) is around 37 kcal/mol, well above that corresponding to isolated O${}_{2}$ (22.6 kcal/mol) [58]. On the bottom panel, the open-shell singlet energies were calculated using the broken-symmetry DFT approach. Around the singlet minimum, singlet and open-shell singlet energies are equal, as expected. However, for large r${}_{CO}$ and/or r${}_{\mathrm{HT}}$ the open-shell singlet energies are significantly smaller. In particular, at the geometries corresponding to the reactants asymptote, the energy difference between the singlet and the triplet state is significantly smaller (27 kcal/mol), in better agreement with the experimental energy difference for the ${}^{3}$O${}_{2}$→${}^{1}$O${}_{2}$ transition.

**Figure 3:**
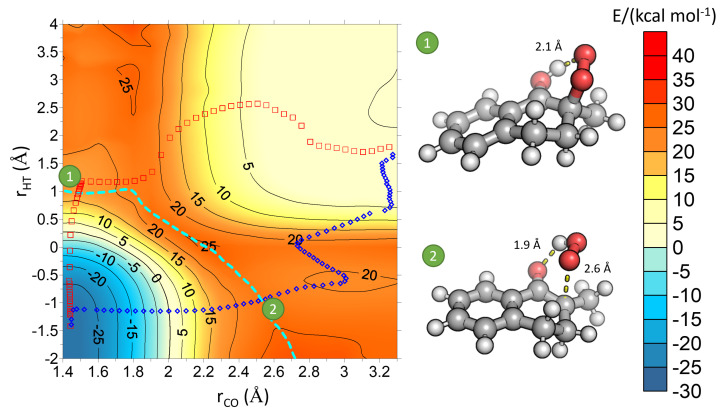
Representation of reaction paths over the 3D potential map, as a function of the energy minimum between the singlet and triplet states. Structures #1 and #2 correspond to the two MECPs obtained, and the red squares represent the geometries of the CO pathway, with the blue diamond showing the geometries of the OOH pathway. The cyan dashed line separates the region in which the singlet and the triplet are more stable. Energies are expressed in kcal/mol, following the color scheme shown in the scale, and distances are expressed in Å. Calculations were carried out at the M062X-D3/maug-cc-pVDZ level of theory. Cartesian coordinates corresponding to the two MECPs are shown in Table S1.

The cuts of the PESs shown in Figure 2 confirm that the reaction proceeds via a transition from the triplet state (only PES where the reactants are stable) to the singlet state (only PES where the peroxide is stable). For each point of the contour plot, the minimum energy between the singlet and triplet states is shown in Figure 3, where the dashed cyan line divides the plot according to the more stable state, with the singlet state being more stable in the bottom-left region, and the triplet state in the upper-right region of the plot. It should be noticed that the dashed line does not correspond to the crossing seam, as the geometries were optimized separately on the triplet and singlet PESs. Starting from different geometries of the contour map, we calculated the MECPs, obtaining two MECPs whose structures are displayed as #1 and #2 in Figure 3, and whose energies are 17.2 kcal/mol and 20.0 kcal/mol, respectively, above the reactants asymptote.

The presence of two TSs on the triplet PES and the two MECPs suggested that, at least, two different mechanisms coexist for this reaction. To characterize them, we calculated the minimum energy paths (MEPs), combining the IRC calculations, starting from the two saddle points, with downhill optimizations from the two MECPs on the triplet and singlet states. An analysis of the MEPs confirmed that each of the TSs on the triplet states was connected with one MECP, which allowed us to define two different mechanisms (see the bottom panel of Figure 1): (i) the CO-pathway, in which the C${}_{\alpha }$O bond is formed before the proton is transferred, and in which the singlet-triplet hop occurs with the CαO1 bond already formed (geometries are shown as red squares in Figure 3); and (ii) the OOH-pathway, in which O${}_{2}$ first picks up the proton of **2** and ISC occurs once the OOH moiety is formed (geometries are shown as blue diamonds in Figure 3). As for both pathways we have a two-step process, we used as the reaction coordinate first, the CαO1 distance and then the r${}_{\mathrm{HT}}$ distance for the CO-pathway, and vice versa for the OOH-pathway. Interestingly, we did not find a mechanism in which CO formation and H-transfer occurs simultaneously.

**Figure 4:**
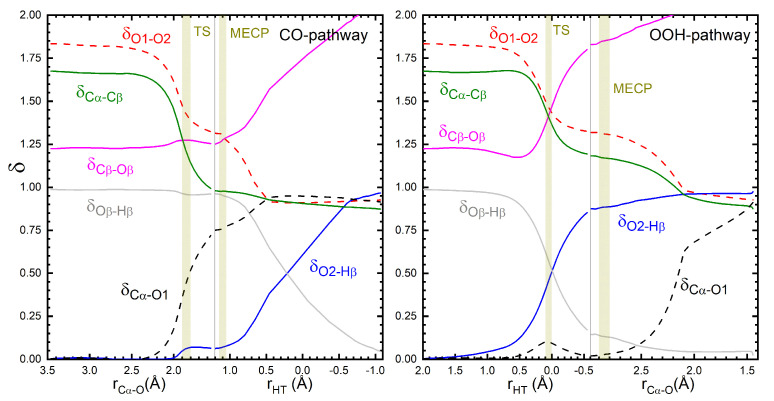
Evolution of delocalization indices along the addition of O${}_{2}$ to 2-methyl-3,4-dihydro-1-naphthol along the reaction path calculated at the M062X-D3/maug-cc-pVDZ level of theory. Results for the CO-pathway and the OOH-pathway are shown in the left and right panels, respectively. The locations of the two TSs and MECPs are shown as vertical shaded lines.

To obtain a quantitative insight into the order in which the bonds are formed/broken throughout the two mechanisms, we calculated the Delocalization Indices (DIs) along the two MEPs. Delocalization indices represent the electron density that is shared between two partners, and it is intimately related to the concept of “bond order”. Unlike internuclear distances, whose evolution can also be used to obtain some insight into the reaction mechanism, DIs depend on the electronic state, and hence, at each point of the MEPs, we represented that corresponding to the ground state. DIs also permit to discern between proton transfer or hydrogen atom (proton+electron) transfer mechanisms. In Figure 4, we display the DIs (δ) for all the bonds that are formed/broken throughout the reaction. At the reactants asymptote, $\delta_{O1-O2}$ is 1.83, which is very close to the value obtained for isolated O${}_{2}$ (1.84), which indicates that the O=O bond is not perturbed by the substrate. This is different from what was observed for the interactions between O${}_{2}$ and the negatively charged enolates, for which $\delta_{O1-O2}$ was somewhat smaller, even at large internuclear distances [36]. Also at the reactants asymptote, $\delta_{C\alpha -C\beta }$ is compatible with a double bond, while $\delta_{C\beta -O\beta }$ and $\delta_{O\beta -H\beta }$ are close to the expected values for single bonds (δ∼1).

For the CO-pathway, the DIs barely change until the two reactants are significantly close to each other (r${}_{C\alpha -O}$ = 2.2 Å). When the reactants are closer, electron density starts to be transferred from the O1−O2 bond to Cα−O bond, and as a consequence of that, $\delta_{C\alpha -C\beta }$ also decreases. At the TS, $\delta_{O1-O2}$ is between the expected value for a single and a double bond, while $\delta_{C\alpha -O1}$ is half of the expected value for a single bond. At the MECP, the latter has raised so we could consider that the Cα−O bond is almost completely formed, while the H atom remains on the substrate. After MECP, the H atom is completely transferred, as evinced for the increase in $\delta_{O2-H\beta }$, which is also associated with the Cα−Cβ double bond and the lack of electron density shared between Oβ and Hβ.

The mechanism for the OOH-pathway is significantly different. First, the H is transferred, and at the TS, the electron density between $\delta_{O2-H\beta }$ is similar to that between $\delta_{O\beta -H\beta }$. It leads to a smaller density shared between O1 and O2, and Cα and Cβ. DIs are compatible with a hydrogen atom transfer instead of a proton transfer, and the formation of a radical pair. To confirm this finding, spin populations are displayed in Figure S2 of the Supplementary information for the two fragments. After the TS, the proton is transferred to O${}_{2}$, and $\delta_{O1-O2}$ and $\delta_{C\alpha -C\beta }$ are between the values expected for a single and a double bond. Once H is transferred, the electron density barely changes with the decrease in Cα−O distance until the MECP is reached and the system swaps to the singlet state. In fact, hopping from the triplet to the singlet state is associated with a significant increase in the electron density shared between $\delta_{C\alpha -O1}$, which is the last bond to be formed in the OOH pathway.

**Figure 5:**
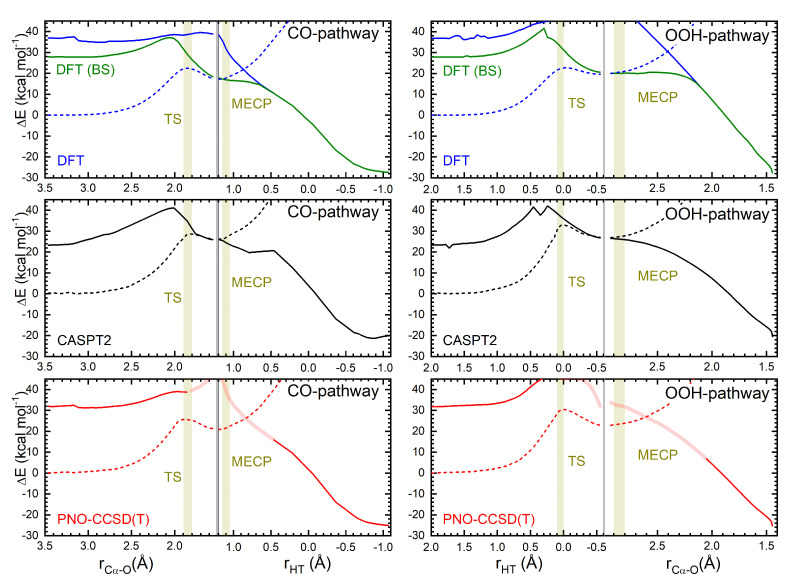
Energy profile for the addition of O${}_{2}$ to 2-methyl-3,4-dihydro-1-naphthol along the two possible reaction paths at M062X-D3 (top panels), CASPT2(8,5) (middle panels), and DLPNO-CCSD(T) (bottom panels). Dashed and solid lines denote triplet and singlet states, respectively. For DLPNO-CCSD(T) calculations, the regions where T1 diagnosis predicts a significant non-monoreference character are shown in brighter red. The basis set employed is maug-cc-pVDZ for all the panels.

Once the main features of the two possible pathways were described, their corresponding reaction energy profiles were calculated at the DFT level of theory and are compared with those obtained using a higher level of theory methods such as DLPNO-CCSD(T), and CASPT2. We selected these two methods because CASPT2 includes both static and dynamic correlations, and is among the most accurate methods for multiconfigurational problems, while Coupled-Cluster methods are considered as the gold standard for the study of single-reference systems. Regardless of the method used and the pathway, the energy profiles displayed in Figure 5 showed similar features, with DFT predicting somewhat smaller barriers. Within the DFT framework, we calculated the energy of the singlet state both using restricted DFT and a broken symmetry approach. Our results show that the restricted DFT energy profile for the singlet state resembles that obtained using DLPNO-CCSD(T) calculations, while the broken symmetry solution is more similar to that obtained using CASPT2.

For the CO-pathway, the system first has to overcome a barrier of around 22–29 kcal/mol (depending on the level of theory, see Table 1) on the triplet state. As commented above, the singlet state was significantly higher in energy, but contrary to what was observed for spin-forbidden reactions between a negatively charged enolate and molecular oxygen [36], the energy of the singlet state does not decrease with the approach of molecular oxygen and the substrate, and we observe either an energy plateau or even a barrier. For those systems, it was proposed that the reaction could also proceed via direct electron transfer, after which the reaction proceeds barrierless [30]. The presence of an electronic barrier on the singlet state makes direct electron transfer unlikely for this system. Regarding the energy difference between the singlet and the triplet state, an analysis of the asymptotic delocalization indices (Figure 4) revealed that the molecular oxygen molecule was unperturbed at the reactants asymptote, so the energy difference should resemble that for isolated O${}_{2}$. That is the case when the CASPT2 method is used, while monoreference methods such as DFT or DLPNO-CCSD(T) predict a higher energy difference between the singlet and the triplet state.

After the TS, the energy of the triplet state slightly decreases, and between the TS and the MECP it reaches a plateau. At this region, differences between the three methods arise. For CASPT2, the singlet and the triplet states are approximately degenerate in this region, and this degeneracy is only broken when the H is being transferred to the peroxide. This behavior is well accounted for broken symmetry DFT. The main difference between the CASPT2 and DLPNO-CCSD(T) energy profiles is that for the latter, the singlet and triplet states are not approximately degenerate between the TS and the MECP, due to a significant destabilization of the singlet state, and it only crosses at one point, r${}_{\mathrm{HT}}$ = 0.7 Å, close to the MECP predicted from restricted DFT calculations. As commented above, CCSD(T) methods are the gold standard for single-reference methods but they may not be fully reliable for strong-correlated systems. To assess the reliability of the DLPNO-CCSD(T) calculations, we calculated the diagnostic T1 along the reaction path. Values of T1 below 0.02 represent regions that are reasonably described using Coupled-Cluster methods (0.044 for open-shell systems) [59,60]. Regions that are not well described using DLPNO-CCSD(T), according to its T1, are shown as shaded in Figure 5, and for the CO-pathway, DLPNO-CCSD(T) is not fully reliable only for the singlet state and between the TS and the crossing point. As a consequence of that, CCSD(T) calculations around the MECP are not accurate. Regardless of the method used, after reaching the MECP, the singlet state becomes stabilized as expected, and the reaction proceeds barrierless.

Qualitatively, the energy profiles are similar for the OOH pathway, with the difference being that the T1 diagnosis predicts that DLPNO-CCSD(T) not only is not reliable for the singlet state between the TS and the crossing point, but also beyond the MECP. This is not surprising, since DIs indicate that a radical pair was formed in that region.

To obtain a deeper understanding of the nature of the lowest energy states involved in the reaction, energy profiles were also calculated using MRCI based on a state average CASSCF wave-function that treats all the states on equal footing (Figure S3 in the Supplementary Material). Qualitatively, energy profiles for the lowest singlet and triplet states (S1 and T1) are similar to those obtained for CASPT2, although the barriers obtained are significantly larger, as expected due to the limited dynamic correlation included. The reference active space was small, with the dioxygen π orbitals and the p${}_{z}$ orbital for the bonding carbon atom, forming a CAS(4,3) space [27]. These calculations were carried out with Molpro2020 [61]. At the reactants asymptote, the order of the states involved mimics that for molecular oxygen: the ground triplet state (that correlates to the ${}^{3}\Sigma_{g}^{-}$ of O${}_{2}$), two degenerate singlet states (that originate from the ${}^{1}\Delta_{g}$), and a higher energy singlet state (that comes from the ${}^{1}\Sigma_{g}^{+}$ of O${}_{2}$). At higher energies, we obtain two degenerate singlet states that come from charge-transfer ionic pairs between tetralone and O${}_{2}$. Between TS and the MECP, the ground singlet and triplet states are degenerate, as we found for CASPT2 and BS-DFT.

**Figure 6:**
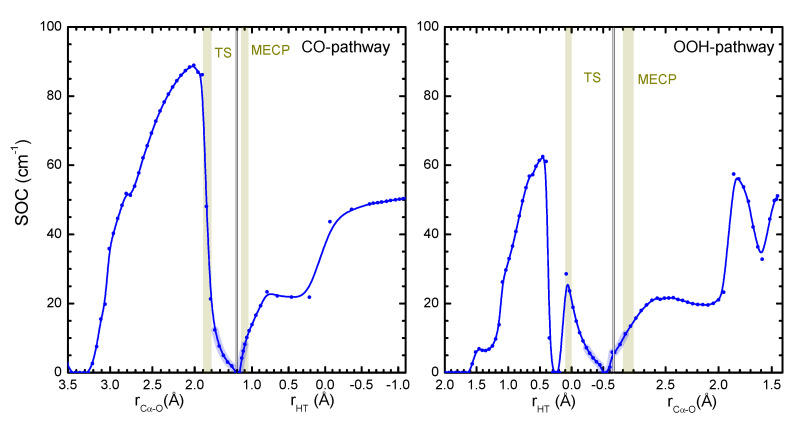
Spin -orbit coupling (SOC) between the ground triplet state and the singlet state involved in the oxidation of 2-methyl-3,4-dihydro-1-naphthol. Calculations carried out at the CASPT2(8,5)/maug-cc-pVDZ theory level. Dots represent the actual values, while the solid lines are displayed to guide the eye. The regions for which singlet and triplet states are almost degenerate are highlighted, as they represent the regions for where hopping is more likely to occur.

According to NA-TST, the determination of the MECP is not enough to characterize the dynamical bottlenecks for spin-forbidden processes, as the hopping probability depends on other factors, such as the SOC. In Figure 6, we calculated the SOC between the lowest energy singlet and triplet states along the reaction path using the CASPT2 method. As SOC depends strongly on the nature of the two states involved, it is expected to be very different for the two pathways in the “hopping” region, where the singlet and triplet states show similar energies. For the CO-pathway, the largest SOC (about 90 cm${}^{-1}$) is found before the TS, when r${}_{C\alpha -O}$ is around 2 Å. As a reference, the spin-orbit coupling value for systems with metal atoms can be between 300 and 1000 cm${}^{-1}$ [38], and the obtained values for other systems involving the cofactorless addition of O${}_{2}$ to the organic substrate are about 70–80 cm${}^{-1}$ [27,32,43]. The SOC value decreases with the approach of the two partners, leading to values even close to zero and rising again at the end of the interaction region, when the peroxide is being protonated. In the region where the singlet and triplet are approximately degenerate, SOC goes from almost null to around 10 cm${}^{-1}$, and together with the difference of the gradients variations, it leads to an additional hindrance of 2.5–4.0 kcal/mol in that region (Figure S4 of the Supplementary Information). In particular, at the MECP, the SOC is ∼10 cm${}^{-1}$, leading to $\Delta \Delta G^{‡}$ = 3.1 kcal/mol. The barrier obtained is in any case lower than the previous triplet TS, so the MECP step would not be the limiting step of this reaction.

For the OOH-pathway, SOC reaches a maximum at around 60 cm${}^{-1}$ at the beginning of the interaction region, after which it drops where the proton is being transferred to the O${}_{2}$ moiety, and a radical pair is formed. As predicted by the El-Sayed rules, this is caused by the similar character of the two involving states, as hopping between singlet and triplet only involves the change of spin of one electron. Once the proton has been fully transferred and the two partners approach, SOC increases, and it obtains a value of about 15 cm${}^{-1}$ at the end of the “hopping” region, where r${}_{C\alpha -O}$ is around 2.4–2.5 Å. Even higher values are obtained at lower distances, although they are not relevant, as singlet and triplet states have considerably different energies in that region. Considering the region where singlet and triplet are almost degenerate, the SOC ranges between 0 and 20 cm${}^{-1}$. However, the lower difference of gradients favors this path, and $\Delta \Delta G^{‡}$ is only 2.0 kcal/mol at MECP. Again, this step does not seem to be limiting to the reaction, and the final kinetics will be determined from the first step, at the triplet TSs.

**Figure 7:**
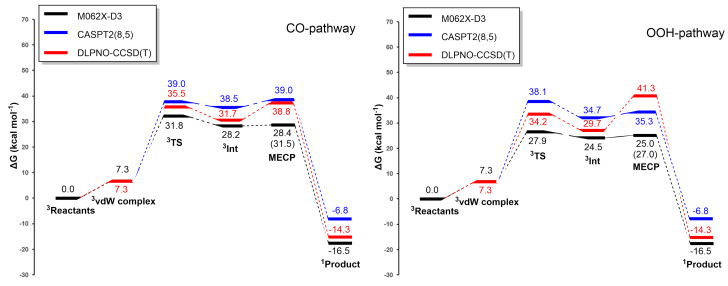
Free energy profile for the addition of O${}_{2}$ to 2-methyl-3,4-dihydro-1-naphthol along the two possible reaction paths for M062X-D3, CASPT2(8,5), and DLPNO-CCSD(T). M062X-D3 and DLPNO-CCSD(T) single-points with maug-cc-pVTZ basis set over maug-cc-pVDZ geometries and vibrational analysis in acetonitrile implicit solvent (SMD) level of theory. CASPT2 single-points with maug-cc-pVDZ basis set. Each structure was optimized for the lowest spin state. At MECP, the increase in free energy caused by the limited hopping probability is shown in parentheses. Left panel: CO−pathway. Right panel: OOH-pathway. Energies (in kcal/mol) refer to the reactants’ asymptote where the two reactants are separated. Energies correspond to the ground state for each geometry. Energies for triplet and singlet states are shown in Figure S5.

In Figure 7, we show the free energies calculated for both paths using an improved basis set and also considering the effect of the solvent (using the SMD implicit solvent model with the dielectric constant of acetonitrile). The free energies of TSs and MECPs are considerably higher than the electronic energies due to entropy penalty [62]. According to Figure 7, the OOH pathway is preferred over the CO-pathway for DFT and CASPT2, while for Coupled-Cluster, the CO-pathway is preferred. Regarding the rate-limiting step, the barriers associated with the TS on the triplet state and the ISC are similar, with the former being the rate-limiting step for the OOH pathway for DFT and CASPT2. The barrier associated with ISC is larger using DLPNO-CCSD(T), and in particular, for the OOH pathway, albeit results should be taken with care due to the high value of the T1 diagnosis. According to our free energy barriers, the reaction should be slow at 298 K, happening over a few days (as described in the experiment, 129 h) [51]). At this point, it is possible to compare the results obtained for this reaction with those obtained for the enzymatic cofactorless of O${}_{2}$ to organic substrates, for which the deprotonation of the substrate is a common preactivation step. When the substrate is deprotonated, there is no barrier on the triplet potential energy surface, and the rate constant of the process depends on the energy of the MECP and the hopping probability, the latter being increased by the negative charge of the system [27,36]. If O${}_{2}$ is added to a neutral substrate, there is a higher barrier on the triplet state, similar to or even higher than that associated with ISC. Hence, a lower hopping probability has a small effect on the rate of the process.

### Phosphite Reduction Mechanism

**Figure 8:**
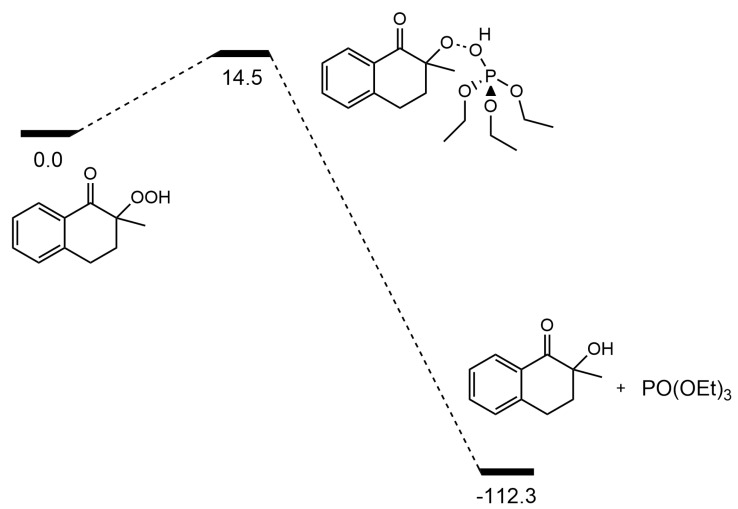
Free energy profile for the reduction of the hydroperoxide to the α−ketol at DLPNO-CCSD(T)/maug-cc-pVTZ//M062X-D3/maug-cc-pVDZ in the ethanol implicit solvent (SMD) level of theory. Energies are given in kcal/mol, and the zero of the energy scale corresponds to the energy of the isolated reactants. Cartesian coordinates of reactants and the transition state are shown in Table S1.

Experimentally, the reduction of the hydroperoxide with P(OEt)${}_{3}$ was very fast, leading to formation of the α-ketol in a few minutes [51]. Our calculations predict that the free energy barrier for the process is just 14.5 kcal/mol at room temperature (Figure 8), considerably smaller than the one obtained for the formation of the peroxide. This free energy barrier is mostly associated with the entropy burden (the electronic barrier is just 2.3 kcal/mol). According to our calculations, the reaction occurs in a single step, in which the phosphorous attacks the hydroxyl oxygen of the hydroperoxide, leading to the breaking of the peroxide bond, which also triggers proton transfer, forming the α-ketol. Proton transfer is favored by interaction with the carbonyl oxygen in α to the peroxide. This mechanism is in good agreement with the proposal of Denney et al. [63] in the 1960s, which suggested a simultaneous breaking of the peroxide and proton transfer. As the reaction was carried out in EtOH, we also carried out calculations with one explicit EtOH molecule that may stabilize the transition state, and observed a small effect. This is not surprising since phosphites can also reduce hydroperoxide in pentane at low temperatures [63].

Although we are not aware of modern mechanistic studies for the reduction of hydroperoxides by phosphites, the oxidation of aromatic substrates by C4a-hydroperoxy-FADH has been widely studied over the last few years. Very recently [64], it has been proposed that the breaking of the C4a-hydroperoxy-FADH peroxide may follow a hydroxyl radical-coupled electron-transfer mechanism with the participation of singlet and triplet states. We also explored this possibility, but in our case, the triplet state was high enough in energy to disregard any role in the reaction.

## 3. Materials and Methods

### 3.1. Calculations for the Peroxidation of 2-Methyl-3,4-dihydro-1-naphtol (***2***)

To investigate the peroxidation mechanism of the enol (**2**), we carried out geometry-restrained optimizations at an M062X-D3/maug-cc-pVDZ [65] level of theory using two reactions coordinates, which were defined as: (a) r${}_{CO}$, the distance between Cα of the enol (see Figure 1) and the closest oxygen atom of O${}_{2}$, and (b) the proton transfer coordinate from Cβ of the enol to molecular oxygen, which was defined as r${}_{\mathrm{HT}}$ = r${}_{O2-H\beta }$ − r${}_{O\beta -H\beta }$. The values of r${}_{\mathrm{CO}}$ range from 1.4 Å to 3.2 Å in steps of 0.1 Å, and the values of r${}_{\mathrm{HT}}$ range from −2 Å to 4 Å in steps of 0.2 Å. Further restraints were added to impede the addition of O${}_{2}$ to Cβ and the formation of an endothermic product (Figure S1 in the Supplementary Material), associated with a higher barrier and which otherwise appears in the contour maps of the singlet PES around (r${}_{\mathrm{HT}}$ = 1 Å, r${}_{\mathrm{CO}}$ = 2.1 Å). Calculations were carried out using Gaussian16 [66] for the two possible multiplicities, singlet and triplet. Stationary points (minima and saddle points) were optimized again without any restraint using the M062X-D3/maug-cc-pVDZ level of theory using Gaussian16 [66], starting from the different geometries extracted from the 3D energy profile. Frequencies were calculated to ensure convergence.

Calculations for the singlet state were repeated using broken symmetry DFT (BS-DFT) [67,68], as implemented in Gaussian16 [66], which introduces a breakdown of the spatial and spin symmetry in decoupled alpha and beta spin-orbital contributions. To correct spin contamination, we used Yamaguchi’s spin projection [69,70] to describe the energies of the open-singlet states:

(1) $${}_{\mathrm{SP}}^{\mathrm{singlet}}E{=}^{\mathrm{singlet}}E+C_{\mathrm{SC}}\left[{}^{\mathrm{singlet}}E\;-{\;}^{\mathrm{triplet}}E\right]$$

where ${}_{\mathrm{SP}}^{\mathrm{singlet}}E$ is the corrected open-shell singlet energy, ${}^{\mathrm{singlet}}E$, the open-shell singlet electronic energy obtained with BS-DFT, and ${}^{\mathrm{triplet}}E$, the triplet electronic energy. The coefficient $C_{SC}$ is calculated as follows:

(2) $$C_{\mathrm{SC}}=\frac{{}^{\mathrm{singlet}}\langle S^{2}\rangle }{{}^{\mathrm{triplet}}\langle S^{2}\rangle \;-{\;}^{\mathrm{singlet}}\langle S^{2}\rangle }$$

where ${}^{\mathrm{singlet}}\langle S^{2}\rangle$ and ${}^{\mathrm{triplet}}\langle S^{2}\rangle$ are the uncorrected expectation values for the total spin angular momentum of the open-shell singlet and triplet, respectively. This methodology should yield similar qualitative results as the more expensive methods employed here [36,71].

The minimum energy crossing points (MECPs) between the singlet and triplet states were calculated using the method developed by Harvey et al. [72]. In this method, an MECP search is performed by following the effective gradient given by the combination of *f* and *g*, which are defined as:

(3) $$\begin{array}{c} f=\left({}_{\mathrm{SP}}^{\mathrm{singlet}}E{-}^{\mathrm{triplet}}E\right)x_{1} \end{array}$$

(4) $$g=\left(\frac{\partial^{\mathrm{singlet}}E}{\partial q}-\frac{x_{1}}{|x_{1}|}\left[\left(\frac{\partial^{\mathrm{singlet}}E}{\partial q}\cdot \frac{x_{1}}{|x_{1}|}\right)\right]\right)$$

where *q* represents the cartesian coordinates, and $x_{1}$ is defined as:

(5) $$x_{1}=\left(\frac{\partial^{\mathrm{singlet}}E}{\partial q}-\frac{\partial^{\mathrm{triplet}}E}{\partial q}\right)$$

The reaction paths connecting stationary points (saddle points and minima) and MECPs were calculated at a M062X-D3/maug-cc-pVDZ level of theory using intrinsic reaction coordinate (IRC) calculations. When the MECPs were used as starting coordinates, calculations were made independently for the singlet and triplet states. Calculations were repeated at the CASSCF/CASPT2 [73,74,75,76,77], and DLPNO-CCSD(T) [78] levels of theory, using a subset of the geometries generated in the IRC calculations. Finally, Gibbs free energies were calculated at the M062X-D3/maug-cc-pVDZ, and energies were refined at M062X-D3/maug-cc-pVTZ in implicit SMD solvent using an acetonitrile dielectric constant [79,80,81,82].

DLPNO-CCSD(T) calculations were performed using Orca5 [83] and a maug-cc-pVDZ basis set. Final single-points were refined with the maug-cc-pVTZ basis set. As the accuracy of the Coupled-Cluster methods is limited to mono-referential regions, a T1 diagnosis was carried out as a threshold for reliability. For mono-referential systems, the accuracy of DLPNO-CCSD(T) is comparable to that of CCSD(T), with differences in energies of less than 0.2 kcal/mol [84]. CASPT2 calculations were carried out using OpenMolcas [85]. We selected an (8,5) active space, including the π and $\pi^{*}$ O${}_{2}$ orbitals and the *p* orbital of Cα perpendicular to the molecular plane.

To shed more light on how the electronic density is rearranged along the pathways, delocalization indices (DIs) [86,87,88] were calculated as implemented in the NDELOC code [89] with the Mulliken partition scheme [90]. For DIs calculations, diffuse functions were not included, to avoid an erroneous definition of the Hilbert space.

### 3.2. Spin-Orbit Coupling and Hopping Probabilities

The spin-orbit couplings (SOCs) were calculated at the CASPT2 level of theory, and the effective SOC was obtained using the expression [39]:

(6) $$H_{SO}^{total}=\sqrt{S_{-1}^{2}+S_{0}^{2}+S_{1}^{2}}$$

where S${}_{M}$ is the coupling between the singlet state and each of the individual M${}_{S}$ components of the triplet state. $H_{SO}^{total}$ was used to calculate the hopping probability between surfaces, using the Landau-Zenner formula [37,39]:

(7) $$p^{LZ}\left(\varepsilon \right)=\exp\left(-\frac{2\pi H_{SO}^{2}}{\hbar |\Delta F|}\sqrt{\frac{\mu }{2(\varepsilon -E_{MECP})}}\right)$$

with μ being the reduced mass associated with the ISC. |ΔF| is the norm of the difference between the gradients in the singlet and triplet states. The latter two variables were computed using the Glowfreq software [91]. The double passage version was used:

(8) $$P_{trans}\left(\varepsilon \right)=\left(1-p^{LZ}\right)+p^{LZ}\left(1-p^{LZ}\right)$$

and the hopping probability, $p^{LZ}$, was calculated upon integration over the energy component perpendicular to the crossing seam, after accounting for the energy distribution using a Maxwell-Boltzmann distribution.

For spin-forbidden reactions, non-adiabatic Transition State Theory (NA-TST) provides the following rate coefficient [37]:

(9) $$k_{\mathrm{NA}}\left(T\right)=p^{LZ}k_{\mathrm{adiabatic}}\left(T\right)$$

where $k_{NA}\left(T\right)$ and $k_{adiabatic}\left(T\right)$ are the non-adiabatic and the hypothetical adiabatic rate coefficients for an analogous spin-allowed reaction, with the latter being given by:

(10) $$k_{\mathrm{adiabatic}}\left(T\right)=\frac{k_{B}T}{h}e^{\frac{-\Delta G^{‡}}{RT}}$$

where $k_{B}$ is the Boltzmann constant and T is the temperature. Through the combination of Equations (9) and (10), it is possible to calculate the hindrance to the reaction caused by the spin-forbiddance of the process, $\Delta \Delta G^{‡}$,

(11) $$\Delta \Delta G^{‡}=-ln\left(p^{LZ}\right)RT$$

which, once added to $\Delta G^{‡}$, provides the free-energy barrier for an analogous spin-allowed reaction. For hopping probabilities of between 10${}^{-4}$–0.1, typical values of $\Delta \Delta G^{‡}$ are between 1–4 kcal/mol [38].

### 3.3. Calculations for Reduction with Triethylphosphine

To elucidate the mechanism for the reduction of the peroxide (**3**) with triethylphosphine, stationary points (minima and saddle points) were optimized at the M062X-D3/maug-cc-pVDZ level, and then single-point calculations were obtained using Orca5 [83] at the DLPNO-CCSD(T)/maug-cc-pVTZ level [65]. Gibbs free energies were calculated by combining electronic energies at a DLPNO-CCSD(T)/maug-cc-pVTZ level of theory, with enthalpy and entropy corrections calculated at the M062X-D3/maug-cc-pVDZ level.

## 4. Conclusions

In this work, we have studied the mechanism of the spin-forbidden peroxidation of a naphthol by molecular oxygen using different single-reference and multi-reference computational methods. According to our calculations, the reaction follows a two-step process, and the most likely mechanism is that in which the first molecular oxygen abstracts the O-H proton, forming a radical pair, and second, it swaps its spin state, leading to the barrierless formation of the peroxide. Contrary to what was found for reactions between O${}_{2}$ and the enolates, the two steps of the process show similar barrier heights: one on the triplet PES, associated with O${}_{2}$ protonation, and that associated with the MECP, which provides the effective barrier for the second step. Once in the singlet state, the protonated peroxide is formed with no barrier. ISC between these states is not favorable, as predicted by the El-Sayed rules, leading to an additional hindrance of 2–3 kcal/mol for the intersystem crossing step, which is not high enough to prevent this reaction.

For the enzymatic cofactorless spin-forbidden addition of O${}_{2}$ to an organic substrate, it is typically found that the organic substrate is deprotonated before the reaction. Our results suggest that the deprotonation of the substrate is a successful strategy as it leads to significantly smaller free energy barriers, and even the approach of O${}_{2}$ to the substrate becomes barrierless. It also leads to significantly larger values of the spin-orbit coupling. We believe that our results are general and can be applied to other spin-forbidden additions of O${}_{2}$ to neutral double bonds.

## Figures and Tables

**Table 1:** Electronic energies of critical points along the reaction paths for the different methods used: M062X-D3 (with broken symmetries in parentheses), CASPT2(8,5), and DLPNO-CCSD(T), using the basis set maug-cc-pVDZ. The energies (in kcal/mol) refer to the reactants complex. The MECP energies for the DLPNO-CCSD(T) method correspond to regions where the singlet is not accurately represented by a monoreference method according to the T1 diagnosis, and are not completely reliable. $\Delta E_{S}^{T}$ O${}_{2}$ refers to the energy difference between the singlet and triplet reactant complex (that is associated with the ${}^{3}$O${}_{2}$→${}^{1}$O${}_{2}$ excitation). The reactants and products are the same for the two pathways.

ΔE CO-pathway	M062X-D3	DLPNO-CCSD(T)	CASPT2(8,5)
Reactant complex (triplet)	0.00	0.00	0.00
$\Delta E_{S}^{T}$ O${}_{2}$	36.9 (27.9)	31.8	23.2
TS (triplet)	22.5	25.7	28.6
Intermediate (triplet)	17.1	20.8	26.1
MECP	22.6 (17.2)	26.2	27.0
Product (singlet)	−27.8	−25.5	−20.5
ΔE OOH-pathway	M062X-D3	DLPNO-CCSD(T)	CASPT2(8,5)
Reactant complex (triplet)	0.00	0.00	0.00
$\Delta E_{S}^{T}$ O${}_{2}$	37.0 (27.9)	31.8	23.2
TS (triplet)	22.7	30.4	32.9
Intermediate (triplet)	19.6	22.5	26.6
MECP	30.5 (20.0)	26.9	27.7
Product (singlet)	−27.8	−25.5	−20.5

## Data Availability

Cartesian coordinates of all relevant structures are deposited in Table S1 of the Supplementary Material. Other data is available upon reasonable request.

## Supplementary Materials

The following supporting information can be downloaded at: https://www.mdpi.com/article/10.3390/ijms24087424/s1.
