# Supplementary material for: Spin-Forbidden Addition of Molecular Oxygen to Stable Enol Intermediates—Decarboxylation of 2-Methyl-1-tetralone-2-carboxylic Acid
Source: Int J Mol Sci. 2023 Apr 18;24(8):7424. doi: 10.3390/ijms24087424 (PMC10138960; doi:10.3390/ijms24087424)
Supplement: Supplementary file 1 [file ijms-24-07424-s001.zip › ijms-2258584-supplementary.pdf]

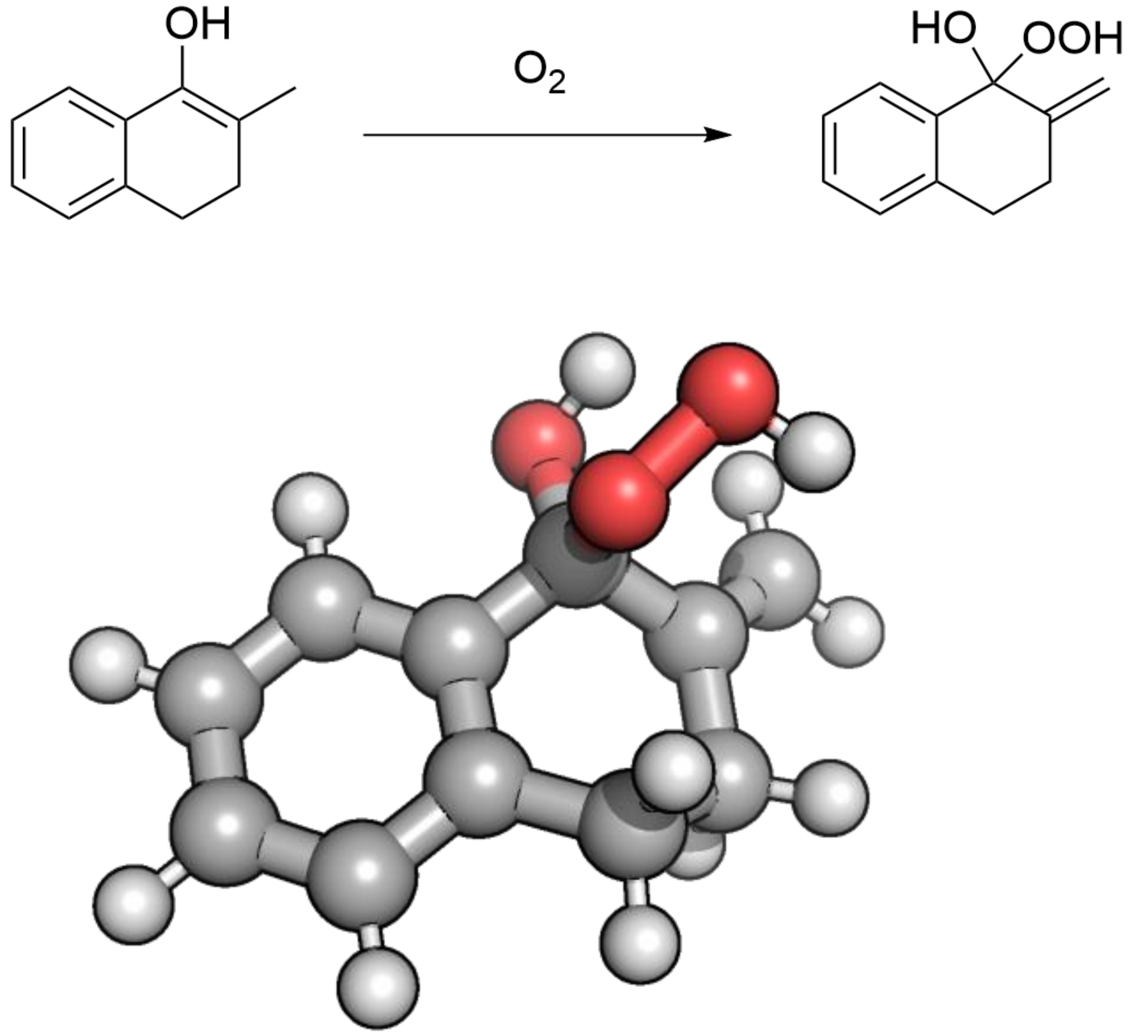

Figure S1. Top panel: Scheme of the side-reaction leading to a non-productive product. Formation of this product is associated with a higher barrier and its electronic energy is 12.9 kcal/mol above that of the products. Bottom panel: Optimized geometry. Cartesian coordinates of the side-product obtained are displayed in the cartesian coordinates section of this supplementary material.

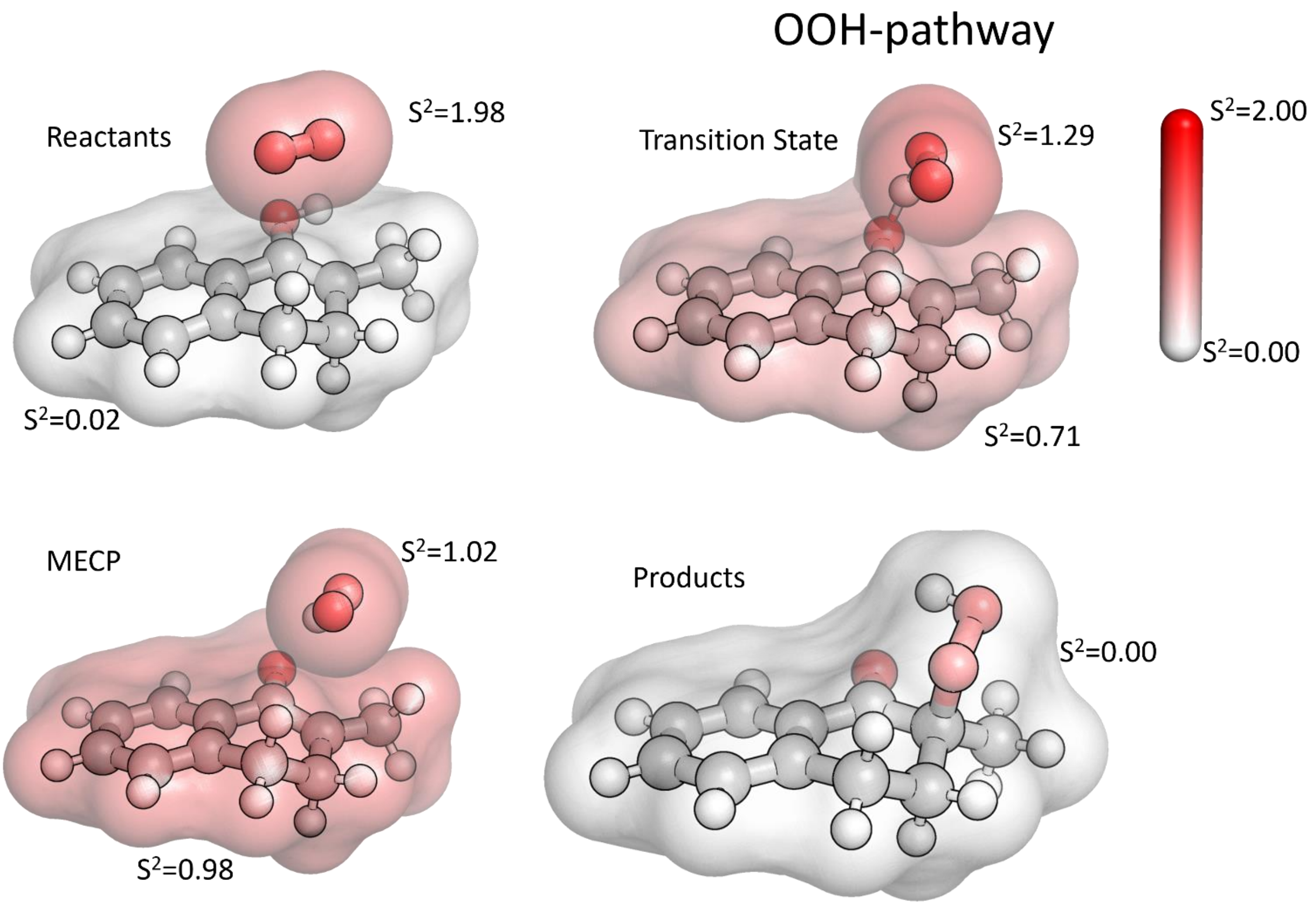

Figure S2: Mulliken Spin populations calculated at the M062X-D3/maug-cc-pVDZ level of theory for the OOH-pathway. Calculations are carried out for the triplet state, except for the products which are optimized at the singlet state. The atoms are displayed in a ball-and-stick representation surrounded by a surface which is coloured using a white-red scheme based on the spin population for each group of atoms. The groups of atoms are defined according to the internuclear distances and at the transition state and MECP one group correspond to the OOH moiety. Mulliken spin populations agree with the DIs calculations supporting that the Hydrogen atom transfer and formation of a radical pair.

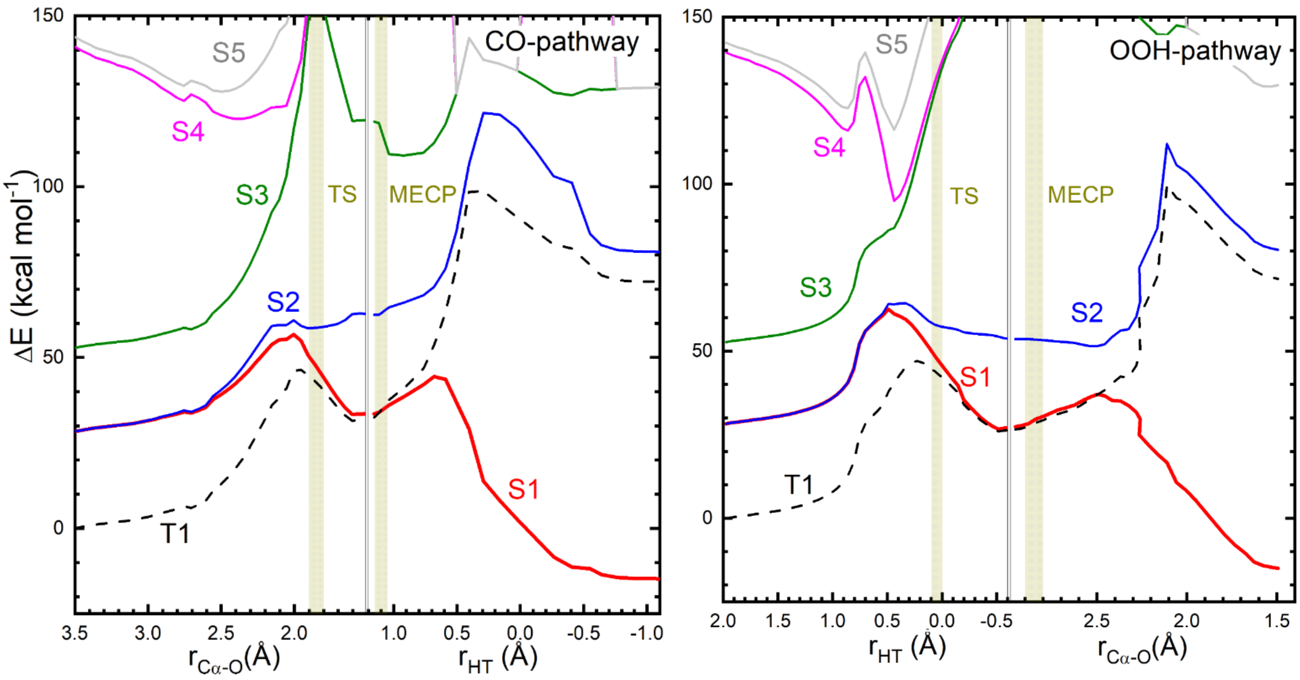

Figure S3. Energy profile for the addition of O_2 to 2-methyl-3,4-dihydro-1-naphthol along the two possible reaction paths for MRCI/6-31+G(d,p) level of theory. Results for the CO-pathway are shown in the top panel and those for the OOH pathway in the bottom panel. Triplet state is shown as dashed lines while the five singlet states are shown as solid lines. The active space is formed by 2 π* antibonding orbitals from O_2 and the p-orbital from Cα (CAS(4,3)).

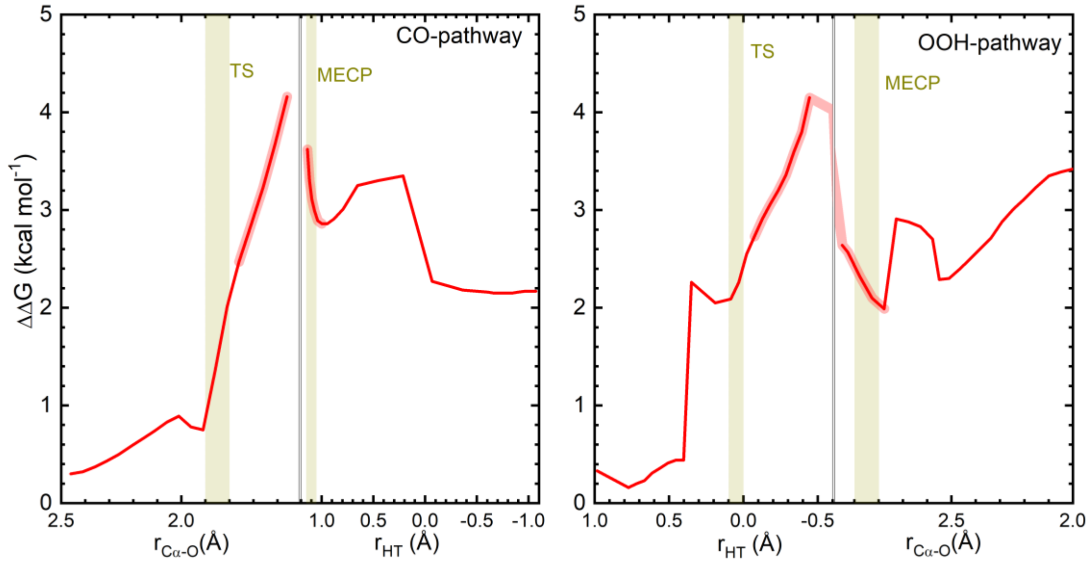

Figure S4. Calculated increase in the free energy profile for the intersystem crossing (ISC) between triplet and singlet, calculating according to Eq. 11 of the main text. Regions in which singlet and triplet states are almost degenerate are red-shaded.

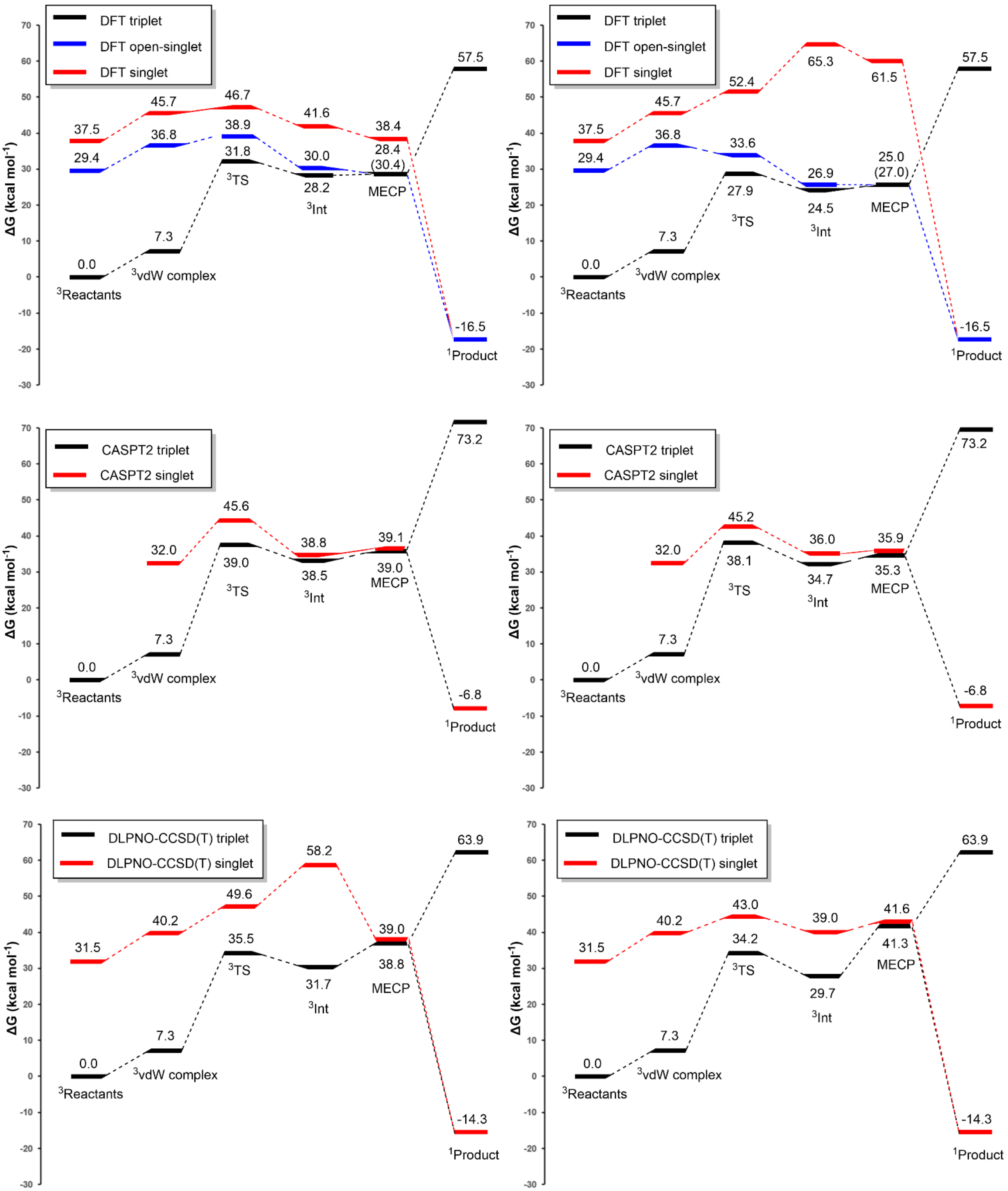

Figure S5. Free energy profile for the addition of O_2 to 2-methyl-3,4-dihydro-1-naphthol along the two possible reaction paths for M062X-D3, CASPT2(8,5) and DLPNO-CCSD(T). Single-points with maug-cc-pVTZ basis sets over maug-cc-pVDZ geometries and vibrational analysis in acetonitrile implicit solvent (SMD) level of theory. Each structure was optimized for the lowest spin state. Increase in free energies due to hopping probability in parentheses for MECP structures. Left panel: CO-pathway. Right panel: OOH-pathway.

Cartesian Coordinates

Table S1. Cartesian coordinates (in Å) for the M062X-D3/ maug-cc-pVDZ optimized geometries for the structures highlighted in Figs 2, 3, and 8 of the main text and Fig. S1 of this supplementary material.

Geometry #1 (Fig.2)

H	1.1969250000	-2.0147750000	-1.4905660000
C	1.5947150000	-1.1928110000	-0.8962280000
C	2.5753290000	0.9194990000	0.6319440000
C	0.7221840000	-0.1996090000	-0.4387790000
C	2.9548590000	-1.1232830000	-0.5977530000
C	3.4481240000	-0.0642240000	0.1627070000
C	1.2133440000	0.8621200000	0.3432190000
H	3.6302860000	-1.8980760000	-0.9621760000
H	4.5120260000	-0.0036950000	0.3953900000
H	2.9573320000	1.7440720000	1.2378330000
C	-0.9141390000	2.1219290000	-0.0829100000
H	-1.6986770000	2.7113700000	0.4162850000
H	-0.5553230000	2.7364770000	-0.9303300000
C	0.2254500000	1.8580110000	0.8957720000
H	-0.1995390000	1.4376860000	1.8257580000
H	0.7355990000	2.7928900000	1.1675270000
C	-0.7152480000	-0.2359780000	-0.7642370000
C	-1.5131800000	0.8382860000	-0.6121400000
C	-2.9637880000	0.8771560000	-0.9966930000
H	-3.6101560000	0.9467400000	-0.1055920000
H	-3.3040120000	0.0091390000	-1.5812160000
H	-3.1681710000	1.7688350000	-1.6114460000
O	-1.2644930000	-1.5758330000	1.8929050000
O	-2.3931400000	-1.5738320000	1.5184320000
O	-1.1176390000	-1.4488210000	-1.2576130000
H	-2.0600080000	-1.4093480000	-1.4606660000

Geometry #2 (Fig.2)

H	-1.5002190000	-2.5171640000	0.1936090000

C	-1.8341760000	-1.4844500000	0.0970150000
C	-2.6491040000	1.1746540000	-0.1604970000
C	-0.8636530000	-0.4558220000	0.0375500000
C	-3.1856850000	-1.1753590000	0.0401770000
C	-3.6026740000	0.1543550000	-0.0813930000
C	-1.2897810000	0.8920830000	-0.1118760000
H	-3.9248870000	-1.9758050000	0.0932200000
H	-4.6650940000	0.3964150000	-0.1214810000
H	-2.9711670000	2.2123010000	-0.2741690000
C	0.9691580000	1.6634610000	0.6157730000
H	1.7594700000	2.4157140000	0.4751390000
H	0.6674230000	1.6887700000	1.6745860000
C	-0.2361000000	1.9553380000	-0.2731640000
H	0.0949560000	1.9832510000	-1.3259540000
H	-0.6515400000	2.9452580000	-0.0393950000
C	0.5279670000	-0.7519050000	0.1088520000
C	1.5723320000	0.2912690000	0.3431530000
C	2.5908730000	-0.1065080000	1.4056940000
H	3.3094450000	0.7118750000	1.5535480000
H	3.1452700000	-1.0076370000	1.1127220000
H	2.0715740000	-0.3095670000	2.3520030000
O	2.3607610000	0.4949680000	-0.9281860000
O	2.8482990000	-0.6109260000	-1.3812670000
O	0.8997090000	-2.0562110000	0.1128990000
H	1.7996710000	-2.1087490000	-0.2491100000

Geometry #3 (Fig.2)

C	3.5924470000	-0.3240360000	0.2890550000
C	2.8071400000	0.7865240000	0.5954680000
C	1.4393910000	0.7893300000	0.3146240000
C	1.6551270000	-1.4670240000	-0.5774400000
C	3.0171500000	-1.4546330000	-0.2967760000
H	4.6607080000	-0.3083230000	0.5103850000
H	3.2600920000	1.6633670000	1.0628270000

H	1.1738270000	-2.3349380000	-1.0296160000
H	3.6328960000	-2.3231300000	-0.5324900000
C	0.8689940000	-0.3472950000	-0.2830650000
C	-0.5833640000	-0.3740750000	-0.6003210000
C	-1.2814170000	0.8820660000	-0.6719550000
C	-0.5613800000	2.1504850000	-0.3441400000
H	-0.1252520000	2.5475230000	-1.2825690000
H	-1.2837680000	2.9046880000	0.0024910000
C	0.5471790000	1.9447740000	0.6882760000
H	0.0830600000	1.7297080000	1.6661140000
H	1.1392980000	2.8626890000	0.8045380000
C	-2.6971990000	0.9393700000	-1.1183670000
H	-3.3492530000	1.1625980000	-0.2554790000
H	-3.0219940000	-0.0084400000	-1.5635590000
H	-2.8344770000	1.7560690000	-1.8450080000
O	-1.1590490000	-1.4618020000	-0.8301250000
H	-2.3790820000	-1.7103110000	0.1982590000
O	-2.5646130000	-0.5626550000	1.6254930000
O	-2.9988960000	-1.6123460000	0.9911260000

Geometry #5 (Fig.2)

H	-1.5104260000	-2.5026830000	0.3416070000
C	-1.8474980000	-1.4779680000	0.1801900000
C	-2.6223860000	1.1651030000	-0.2323680000
C	-0.8833280000	-0.4602450000	0.1389790000
C	-3.1924870000	-1.1748510000	0.0197330000
C	-3.5778140000	0.1536770000	-0.1881210000
C	-1.2630140000	0.8766410000	-0.0695270000
H	-3.9426620000	-1.9652320000	0.0566900000
H	-4.6331710000	0.4017440000	-0.3123200000
H	-2.9313890000	2.2001110000	-0.3924690000
C	0.9987170000	1.6262980000	0.7111400000
H	1.7756730000	2.3972170000	0.6048360000
H	0.7114600000	1.5808740000	1.7741080000

C	-0.2225320000	1.9649560000	-0.1386290000
H	0.0903720000	2.0967570000	-1.1868930000
H	-0.6626380000	2.9176060000	0.1881860000
C	0.5492590000	-0.8386200000	0.3044170000
C	1.6099320000	0.2795990000	0.3386910000
C	2.7877420000	-0.0821210000	1.2232070000
H	3.5731360000	0.6778400000	1.1076080000
H	3.1913520000	-1.0642120000	0.9563440000
H	2.4634580000	-0.1087320000	2.2734670000
O	2.0018020000	0.4503400000	-1.0415230000
O	2.8070590000	-0.6311020000	-1.4705360000
O	0.9026470000	-2.0037040000	0.3208170000
H	2.1632260000	-1.3503820000	-1.6075030000

TS for the CO-pathway

H	1.4930590000	-2.5089490000	-0.2576560000
C	1.8329010000	-1.4813410000	-0.1300980000
C	2.6578500000	1.1633600000	0.2053050000
C	0.8799560000	-0.4468430000	-0.1000850000
C	3.1861240000	-1.1873950000	-0.0071060000
C	3.6032140000	0.1358640000	0.1540950000
C	1.2983290000	0.8908370000	0.0858510000
H	3.9208090000	-1.9927990000	-0.0381680000
H	4.6646770000	0.3687200000	0.2471670000
H	2.9827820000	2.1958250000	0.3512140000
C	-0.9595930000	1.6888530000	-0.6667530000
H	-1.7719750000	2.3968650000	-0.4443970000
H	-0.6872540000	1.8294350000	-1.7280750000
C	0.2443660000	1.9581570000	0.2314190000
H	-0.0975590000	1.9710160000	1.2806390000
H	0.6696210000	2.9483020000	0.0174890000
C	-0.5279160000	-0.7278730000	-0.2739210000
C	-1.4909690000	0.2858900000	-0.5092260000
C	-2.7058640000	-0.0571990000	-1.3351990000

H	-3.4163150000	0.7803750000	-1.3278380000
H	-3.2274250000	-0.9459160000	-0.9535860000
H	-2.4020090000	-0.2594200000	-2.3737250000
O	-2.3503970000	0.4419470000	1.1267280000
O	-2.8018590000	-0.6718790000	1.4962130000
O	-0.9004250000	-2.0270060000	-0.2229170000
H	-1.8173480000	-2.0618010000	0.1010550000

TS for the OOH-pathway

C	3.5187400000	-0.1810480000	0.1961940000
C	2.6972620000	0.9003170000	0.5162990000
C	1.3253970000	0.8477700000	0.2697100000
C	1.6055930000	-1.4032880000	-0.6175200000
C	2.9729920000	-1.3357530000	-0.3686280000
H	4.5905410000	-0.1227950000	0.3914180000
H	3.1263040000	1.7964140000	0.9695610000
H	1.1514940000	-2.2919990000	-1.0567010000
H	3.6155080000	-2.1817250000	-0.6148360000
C	0.7829170000	-0.3133180000	-0.3099290000
C	-0.6658990000	-0.3809480000	-0.5968360000
C	-1.4178460000	0.8070360000	-0.6457190000
C	-0.7538190000	2.1127130000	-0.3339630000
H	-0.3684580000	2.5329770000	-1.2839840000
H	-1.5032700000	2.8269770000	0.0383030000
C	0.3907750000	1.9602820000	0.6668840000
H	-0.0381620000	1.7175740000	1.6547690000
H	0.9407500000	2.9054160000	0.7715630000
C	-2.8521810000	0.8011210000	-1.0403720000
H	-3.4814710000	1.0148890000	-0.1588730000
H	-3.1544920000	-0.1643300000	-1.4622020000
H	-3.0455280000	1.6011960000	-1.7727690000
O	-1.2060260000	-1.5307590000	-0.8363170000
H	-1.8920530000	-1.7899760000	0.1352300000
O	-2.0728640000	-0.5865980000	1.6381030000

O	-2.4167050000	-1.7243820000	1.1946880000

MECP for the CO-pathway. Geometry #1 (Fig.3)

H	-1.5369524000	-2.4528385000	0.2545103000
C	-1.8548055000	-1.4106036000	0.2335591000
C	-2.6287496000	1.2733243000	0.1642661000
C	-0.8724461000	-0.3988476000	0.1129978000
C	-3.1964629000	-1.0721105000	0.3366251000
C	-3.5916569000	0.2694442000	0.3129566000
C	-1.2795722000	0.9616742000	0.0544643000
H	-3.9443511000	-1.8592141000	0.4411550000
H	-4.6457859000	0.5339491000	0.4026968000
H	-2.9363286000	2.3207049000	0.1223412000
C	1.0529437000	1.6637424000	0.5796454000
H	1.8448244000	2.4032854000	0.3884190000
H	0.8470397000	1.6665208000	1.6616422000
C	-0.2223193000	2.0054556000	-0.1853474000
H	0.0077237000	2.0504797000	-1.2644504000
H	-0.5897079000	2.9984432000	0.1093524000
C	0.5116465000	-0.7234855000	0.0394273000
C	1.5983198000	0.2860010000	0.2229614000
C	2.6799490000	-0.1609885000	1.1995452000
H	3.4314782000	0.6342191000	1.3081885000
H	3.1840949000	-1.0737246000	0.8566116000
H	2.2210817000	-0.3614919000	2.1775403000
O	2.2789259000	0.4966590000	-1.1045529000
O	2.7739575000	-0.5975690000	-1.5822376000
O	0.8519811000	-2.0296217000	-0.0773814000
H	1.7305937000	-2.0765336000	-0.4908792000

MECP for the OOH-pathway. Geometry #2 (Fig.3)

C	-3.6044291000	0.3013736000	0.1067626000
C	-2.6440269000	1.3124680000	0.0578038000
C	-1.2851002000	1.0020799000	0.1210706000
C	-1.8659882000	-1.3638216000	0.2271656000

C	-3.2177958000	-1.0384022000	0.1862676000
H	-4.6634174000	0.5596950000	0.0801750000
H	-2.9515270000	2.3550234000	-0.0327310000
H	-1.5279186000	-2.3994425000	0.2673272000
H	-3.9730054000	-1.8244902000	0.2152977000
C	-0.9037177000	-0.3465171000	0.2186365000
C	0.5354000000	-0.6974142000	0.3189325000
C	1.4408942000	0.3238368000	0.7811634000
C	0.9520067000	1.7215177000	0.9706502000
H	0.6403772000	1.8288586000	2.0284843000
H	1.7882057000	2.4218713000	0.8231789000
C	-0.2087606000	2.0527467000	0.0348998000
H	0.1796662000	2.0832836000	-0.9981597000
H	-0.6210967000	3.0466727000	0.2525330000
C	2.8317294000	-0.0130815000	1.1658019000
H	3.5360416000	0.6445897000	0.6305190000
H	3.0629378000	-1.0629068000	0.9619293000
H	2.9678954000	0.1799330000	2.2453922000
O	0.9301051000	-1.8512323000	0.0483446000
H	2.0378286000	-1.7088623000	-1.1228039000
O	2.3319614000	-0.0837469000	-1.9445345000
O	2.6847021000	-1.3288256000	-1.8013715000

TS for the $P(OEt)_3$ reduction reaction (Fig. 8)

C	-2.71552600	2.27889800	-0.21786000
C	-3.91103400	1.70655000	0.54089600
C	-4.17032500	0.25255300	0.23014000
C	-3.19525300	-0.54547800	-0.39415800
C	-1.88968000	0.04225500	-0.78750100
C	-1.47824700	1.36001500	-0.12485700
H	-6.16846800	0.26982900	1.03836600
H	-2.96851800	2.40820000	-1.28269200
H	-3.74771300	1.80501000	1.62720200
C	-5.39458900	-0.33964800	0.56606800

C	-3.44015800	-1.90117300	-0.66599900
C	-4.65430600	-2.47478200	-0.31307200
C	-5.63429800	-1.68564400	0.30209000
H	-2.65967800	-2.49413200	-1.14683200
H	-4.84430200	-3.52983700	-0.51511600
H	-6.59515600	-2.12664400	0.57453300
H	-4.81369600	2.29193900	0.31274100
H	-2.43943000	3.26341800	0.18662900
O	-1.17369400	-0.50459300	-1.62136600
C	-1.07606300	1.10793200	1.33531400
H	-0.12741600	0.55555100	1.37064000
H	-0.93891800	2.08106000	1.83232100
H	-1.83256600	0.53400100	1.89013000
O	-0.45797400	1.98623600	-0.84064900
O	0.93608900	0.91034600	-0.86543900
H	0.42322700	0.23406900	-1.37996200
P	2.56014900	-0.07561300	-0.33101000
O	2.12177100	-0.68945400	1.14701700
O	3.37207900	1.24601100	0.19752700
O	3.85250700	-1.00894300	-0.75302400
C	4.99219400	-1.16542000	0.13648200
C	5.75975700	-2.38682400	-0.30920100
H	4.63050500	-1.27191600	1.17074800
H	5.60605500	-0.25579900	0.06806500
H	6.64128900	-2.51952400	0.33550000
H	5.13665200	-3.29037800	-0.23754200
H	6.10305900	-2.27344600	-1.34789300
C	1.73401500	-2.07819300	1.25008400
C	0.41439100	-2.37712400	0.56885800
H	1.67074000	-2.26552300	2.33004800
H	2.54157200	-2.70692100	0.84079300
H	0.15199500	-3.43100300	0.74628700
H	-0.39064100	-1.74671100	0.97527100

H	0.47523700	-2.22143600	-0.51919500
C	2.68482500	2.37228700	0.79726200
C	2.60863100	3.52428600	-0.17827000
H	3.28210200	2.63889800	1.68019900
H	1.68595800	2.06371200	1.13730500
H	2.12105600	4.38305100	0.30775200
H	3.61751200	3.83057400	-0.49233600
H	2.02509100	3.24839100	-1.06800600

α-ketol product (Fig. 8)

C	1.44654400	1.36774500	-0.70536400
C	0.12836500	1.92827700	-0.17747900
C	-0.95857400	0.88130900	-0.08591300
C	-0.65151600	-0.48993300	-0.05362800
C	0.76102900	-0.95361600	-0.13521900
C	1.85934800	0.09523100	0.03360100
H	-2.56230400	2.32034000	-0.05977700
H	1.35446200	1.12057100	-1.77526400
H	0.28077500	2.36505400	0.82441800
C	-2.30529300	1.25940600	-0.02416600
C	-1.66852600	-1.45110500	0.03411900
C	-2.99757400	-1.05789400	0.10355000
C	-3.31326800	0.30487700	0.07315900
H	-1.38337400	-2.50395700	0.04688600
H	-3.78949700	-1.80378400	0.17660200
H	-4.35590700	0.62342500	0.11935100
H	-0.21730800	2.75281800	-0.81796500
H	2.25553100	2.10697100	-0.60880400
O	1.05989900	-2.12095600	-0.30348100
C	2.05543200	0.32405200	1.53976900
H	2.41483500	-0.60663100	2.00151200
H	2.81795900	1.10333600	1.68521100
H	1.12673600	0.62568200	2.04631000
O	3.05631900	-0.37958200	-0.53578600

H	2.99255000	-1.34962500	-0.53891000

Geometry of the side product. Geometry #1 (Fig.S1)

C	3.42679700	-0.02635100	-0.02031200
C	2.57469700	1.03288700	0.28880800
C	1.19141900	0.85944900	0.26464800
C	1.52018000	-1.45764000	-0.39219300
C	2.90071300	-1.27061700	-0.36283400
H	4.50205700	0.12208500	0.00176400
H	2.98567300	2.00465900	0.54977400
H	1.08761200	-2.41573900	-0.65683400
H	3.56368800	-2.09435600	-0.60698800
C	0.67418100	-0.39591900	-0.08293800
C	-0.82829500	-0.56568300	-0.07454800
C	-1.52728600	0.63275500	-0.71911800
C	-0.89141200	1.99233400	-0.48646200
H	-1.66172600	2.72326900	-0.22024800
H	-0.45400500	2.32301200	-1.43635900
C	0.22264500	1.97359100	0.56966300
H	0.74344000	2.93533500	0.57855400
H	-0.20856500	1.82257800	1.56547100
C	-2.63749000	0.47671600	-1.44061200
H	-2.97579700	-0.01587900	1.28238200
H	-3.04146400	-0.50666100	-1.65651600
H	-3.14823900	1.33405400	-1.87141600
O	-1.18903100	-1.78648200	-0.62439400
H	-2.01890400	-2.05176500	-0.20124500
O	-2.54280000	-0.86725200	1.46449000
O	-1.15950100	-0.55748200	1.33578600
